# Bedtime Procrastination Partially Mediates the Impact of Personality Characteristics on Daytime Fatigue Resulting From Sleep Deficiency

**DOI:** 10.3389/fnins.2021.727440

**Published:** 2021-09-24

**Authors:** Radoslawa Herzog-Krzywoszanska, Beata Jewula, Lukasz Krzywoszanski

**Affiliations:** ^1^Institute of Psychology, Faculty of Pedagogy and Psychology, Pedagogical University of Krakow, Krakow, Poland; ^2^St. Luke’s Provincial Hospital in Tarnow, Tarnow, Poland

**Keywords:** bedtime procrastination, daytime fatigue, Big Five Personality Aspects, self-esteem, self-efficacy, locus of control, mediation, sleep deficiency

## Abstract

Getting good and sufficiently long sleep at night is important for health, effective functioning, and well-being. However, insufficient or delayed sleep are important and growing social problems that can lead to fatigue, poor performance, deterioration of well-being, circadian rhythm disturbances, and health problems. One of the significant determinants of sleep deprivation is bedtime procrastination, which is understood as the individual tendency to postpone going to bed in the absence of any external circumstances that force one to do so. Nowadays, this phenomenon is widespread in various social groups, especially among students. Despite the high prevalence of bedtime procrastination, its relationship with personality characteristics has not yet been thoroughly studied. The presented research aimed to identify the possible impact of the basic dispositional personality traits and trait-like personality characteristics on bedtime procrastination and daytime fatigue resulting from a deficiency of sleep at night. The responses from 399 university students who voluntarily took part in an internet survey were analyzed. The severity of bedtime procrastination was assessed using the Bedtime Procrastination Scale. Five basic dispositional personality traits (extraversion, neuroticism, conscientiousness, agreeableness, and openness/intellect) and their components (aspects) were measured using the International Personality Item Pool – Big Five Aspects Scale. Self-esteem and general self-efficacy were assessed using the Rosenberg Self-Esteem Scale and the General Self-Efficacy Scale. Perceived locus of control was measured using the Delta Questionnaire. The direct and indirect relationships between personality variables and daytime fatigue were investigated using linear regression models with bedtime procrastination as a mediator variable. Industriousness and orderliness, both of which are aspects of conscientiousness, were found to be indirectly associated with daytime fatigue as a consequence of their impact on bedtime procrastination. Volatility and withdrawal, both of which are aspects of neuroticism, were found to be directly related to daytime fatigue without the intermediary impact of bedtime procrastination. Self-esteem was shown to be associated with experiencing daytime fatigue, both directly and indirectly through bedtime procrastination. General self-efficacy and external locus of control were associated with daytime fatigue only directly, without the intermediary role of bedtime procrastination. The results of our research indicate that personality factors may not only play an important role in shaping sleep-related health behaviors, but they also affect well-being during the day.

## Introduction

Procrastination is a voluntary, irrational delay in performing an intended activity despite awareness of the negative effects of postponing it (Steel, 2007). The irrationality of this phenomenon lies in the fact that there are no important obstacles that would prevent the performance of a planned task, yet the procrastinating person still postpones it. The stability of procrastination over time indicates that it should be considered to be a personality trait (Elliot and Thrash, 2002). This phenomenon is commonly widespread among the population (Ferrari et al., 2005), even though most people do not intend to procrastinate. Procrastination can apply to various areas of life, such as academic procrastination in students or procrastination in the workplace. A type of procrastination that was distinguished only relatively recently is bedtime procrastination, as described by Kroese et al. (2014). It consists in delaying the moment of going to bed, even though there are no serious reasons for this. Kroese et al. (2014) showed a significant correlation between bedtime procrastination and general procrastination. Moreover, different studies have shown that there is a relationship between bedtime procrastination and poor self-regulation on one hand, and a lower number of sleep hours and fatigue during the day on the other hand (Kroese et al., 2014, 2016; Kadzikowska-Wrzosek, 2018b; Herzog-Krzywoszanska and Krzywoszanski, 2019). Bedtime procrastination is also associated with a person’s chronotype: higher eveningness is accompanied by greater bedtime procrastination (Kroese et al., 2014; Kadzikowska-Wrzosek, 2018a). Bedtime procrastination is widespread among young people (Kroese et al., 2016; Kadzikowska-Wrzosek, 2018a). As many studies indicate that sleep deprivation and fatigue have negative consequences for health, well-being, and cognitive functioning (Strine and Chapman, 2005; Eller et al., 2006; Ming et al., 2011), further exploration of this topic seems to be important. Determining the correlates between bedtime procrastination and fatigue could help in the development of new opportunities to reduce this problem and thus increase people’s well-being.

### Personality Characteristics, Bedtime Procrastination, and Sleep

Personality characteristics are factors that influence psychophysical health, an important component of which is sleep quality. The results of numerous studies have shown that basic dispositional traits and trait-like personality variables such as self-esteem, general self-efficacy and locus of control are related to sleep outcomes (Kim et al., 2015). The five-factor model (FFM) is the most widely researched and recognized theory that describes patterns in behavior and hierarchically organizes them into five broad dimensions of the human personality. In FFM, these dimensions are defined as openness to experience, conscientiousness, extraversion, agreeableness, and neuroticism (Guilford, 1954; McCrae and John, 1992). Aspects of the five main dimensions are often distinguished in the personality structure. In their concept, DeYoung et al. (2007) distinguished 10 personality sub-dimensions: two for each basic trait. The individual factors within the main dimension represent one of two distinct but related manifestations of the same basic trait. Among the characteristics of the big five, low conscientiousness and high neuroticism are consistently associated with poor quality of sleep and greater feeling of fatigue the next day (Gray and David, 2002; Williams and Moroz, 2009; Kim et al., 2015). However, although numerous studies have shown a relationship between conscientiousness, neuroticism, and tiredness or fatigue, the nature of this relationship is not yet fully understood (Duggan et al., 2014). To better understand the determinants of daytime fatigue resulting from insufficient sleep at night, it also seems important to investigate its relationships with personality characteristics, which are part of core self-evaluation and self-concept, the components of which are self-esteem, general self-efficacy, and locus of control (Judge et al., 1998). Self-esteem is defined as a way of perceiving and evaluating oneself and one’s abilities (Hewitt and Goldman, 1974); self-efficacy includes one’s assessment of one’s ability to act and cope in many situations (Bandura, 1989); locus of control refers to personal beliefs concerning to what extent one’s outcomes are dependent on one’s skill, efforts, or behavior (Rotter, 1966). Core self-evaluation is related to the quality of life and psychophysical health, including sleep (Tsaousis et al., 2007).

Low conscientiousness, high neuroticism, low self-esteem, low general self-efficacy, and external locus of control are all associated with procrastination, which negatively affects sleep outcomes (Kroese et al., 2014; Hairston and Shpitalni, 2016). To our knowledge, there has been no research on the possible mediating role of bedtime procrastination between personality characteristics and fatigue. Combining research on personality traits, bedtime procrastination and fatigue may be a good way to develop effective interventions for and a more comprehensive approach to the problems of bedtime delay and fatigue. It is increasingly important to tailor sleep-quality interventions to individual needs and abilities (van Dongen et al., 2005). In this situation, it seems important to identify individual differences related to fatigue and the characteristics that may mediate this relationship.

In the research model we adopted, personality traits are treated as explanatory variables because they do not change substantially in adulthood (Costa and McCrae, 1994) and influence a wide range of various behaviors (McCrae and Costa, 1999). The experience of fatigue during the day was the response variable since it is an important subjective consequence of sleep deprivation, poor sleep quality, and other sleep problems. Bedtime procrastination was included as a mediating variable because changeable sleep habits were considered a possible link in the relationship between personality traits and the consequences of sleep deprivation experienced during a given period.

## Materials and Methods

### Subjects

We analyzed answers given to questions in an anonymous online survey of 399 university students (357 females) aged from 19 to 27 years (mean age: 21.7; standard deviation: 1.83). The respondents were students in various academic fields and disciplines at the Pedagogical University of Kraków at bachelor’s and master’s levels. They participated voluntarily and received credit points for a voluntary academic course involving participation in research as subjects. All respondents gave informed consent for their participation in the survey and were allowed to withdraw from the survey without providing any explanations. To ensure anonymity, the responses were recorded in a way that does not allow the identification of respondents.

### Measures

#### Bedtime Procrastination Scale

Bedtime procrastination, which is regarded as an individual’s propensity to delay bedtime without proper justification, was assessed using the Polish version (Herzog-Krzywoszanska and Krzywoszanski, 2019) of the Bedtime Procrastination Scale (BPS), originally developed by Kroese et al. (2014). The BPS is a nine-item self-report questionnaire. It consists of four positively worded statements and five negatively worded statements. The subject is asked to rate each statement on a five-point Likert scale: from 1 = “(almost) never” to 5 = “(almost) always.” The BSP score is computed as the average of ratings of all items after reverse coding of negatively worded items; high values reflect greater bedtime procrastination. Herzog-Krzywoszanska and Krzywoszanski (2019) confirmed that the Polish version of BPS has good internal consistency (Cronbach alpha from 0.83 to 0.86) and satisfactory temporal stability in a 10-week test-retest (Pearson correlation for test-retest: 0.68).

#### Big Five Aspects Scale

The five basic domains of personality were assessed using the Polish version (Strus et al., 2012) of the International Personality Item Pool – Big Five Aspects Scale (IPIP-BFAS), developed by DeYoung et al. (2007). IPIP-BFAS provides scores for each of the Big Five domains of personality as well as two distinct aspects within each domain: extraversion (enthusiasm and assertiveness), agreeableness (compassion and politeness), conscientiousness (industriousness and orderliness), neuroticism (withdrawal and volatility), and openness/intellect (intellect and openness). IPIP-BFAS contains 100 statements (20 per each domain, 10 per each aspect) that are rated on a 5-point agreement scale ranging from 1 (completely incorrectly describes me) to 5 (describes me perfectly well). For the Polish version of IPIP-BFAS, the values of Cronbach’s alpha for the ten scales that measure aspects of personality range from 0.72 to 0.88 (Strus et al., 2012).

#### Rosenberg Self-Esteem Scale

The overall level of self-esteem was measured using the Rosenberg Self-Esteem Scale (SES) (Rosenberg, 1965), as adapted to Polish by Łaguna et al. (2007). SES is a widely used and well-validated measure of global self-esteem that reflects one’s overall evaluation of oneself. It consists of 10 statements rated on a four-point scale from “I strongly agree” to “I strongly disagree.” The summed total score on SES ranges from 10 to 40 points: high values reflect high levels of self-esteem. The Polish version of BPS has good internal consistency (Cronbach alpha: 0.83) and moderate temporal stability (Pearson correlation for test-retest: 0.50) in a 1-year test-retest (Łaguna et al., 2007).

#### General Self-Efficacy Scale

General self-efficacy was assessed using the Polish version (Juczyński, 2000) of the General Self-Efficacy Scale (GSES) by Schwarzer and Jerusalem (1995). GSES consists of 10 statements, for which the possible answers are: 1 – Not at all true, 2 – Hardly true, 3 – Moderately true, 4 – Exactly true. The possible score that can be obtained ranges from 10 to 40. The higher the score, the higher the general self-efficacy. The scores on GSES have a unidimensional structure and are configurally equivalent across many different cultures and languages (Leganger et al., 2000; Scholz et al., 2002). In the Polish version of GSES, the correlation coefficients between individual items and the overall score are high and range from 0.47 to 0.63, while Cronbach’s alpha is 0.85. The temporal stability of the Polish version of GSES, assessed by Pearson correlation for the results of a 5-week test-retest, was 0.78 (Juczyński, 2000).

#### Delta Questionnaire

Locus of control was assessed using the Delta Questionnaire, a self-report tool that was originally developed in Polish by Drwal (1980). It consists of two scales: the locus of control (LOC) scale (14 items in total, including 5 negatively worded), and the control “lie” scale (10 items). Since possible responses to the items of the Delta Questionnaire are “True” and “False,” the sum of the points that can be obtained on the LOC scale ranges from 0 to 14. Higher scores on the LOC scale indicate a more external locus of control. Cronbach’s alpha of 0.83 confirms that the scores on the LOC scale exhibit satisfactory internal consistency. Test-retest reliability for 7 months assessed by Pearson’s correlation was equal to 0.51; this indicates moderate temporal stability of the LOC scale (Drwal, 1980).

### Statistical Analysis

Means with 95% confidence intervals and medians were used as measures of the central tendency of the scores obtained on scales of psychometric tools. The standard deviations and interquartile ranges were applied as dispersion measures of the scores. A series of mediation analyses with multiple outcomes in the structural equation modeling approach (Iacobucci, 2008) was performed to verify whether bedtime procrastination mediates the impact of personality variables on self-reported frequency of experiencing a sense of tiredness during the day. In the analytic model we adopted, the answers given to the question about the average frequency of experiencing daytime fatigue during the week was the response variable and scores in the Bedtime Procrastination Scale was the mediating variable. In the first mediation analysis, the scores on the IPIP-BFAS scales, which measure the major personality domains of the Big Five model of personality (Neuroticism, Agreeableness, Conscientiousness, Extraversion, and Openness/Intellect), were entered as explanatory variables. In the second mediation analysis, the scores in those subscales of IPIP-BFA, which measures both aspects of domains showing statistically significant direct or indirect relationships with the dependent variable in the first model were entered as explanatory variables. To reduce the number of parameters in the regression models, the scores on the self-evaluation and self-concept scales (RSES, GSES, and LOC) were included as explanatory variables in the separate mediation analysis.

Since the level of measurement of the self-reported, averaged frequency of experiencing daytime fatigue was ordinal, the diagonally weighted least squares (DWLS) estimation method was applied. Standardized parameter estimates for indirect, direct, and total effects were computed and tested for significance using bias-corrected percentile bootstrap confidence intervals (Bollen and Stine, 1990; Biesanz et al., 2010) with 5,000 replications. JASP’s (JASP Team, 2020) SEM module, which is based on the lavaan R package for structural equation modeling (Rosseel, 2012), was used for analyses. The R code with the lavaan syntax defining the mediation models is attached as Supplementary Material.

## Results

**FIGURE 1:**
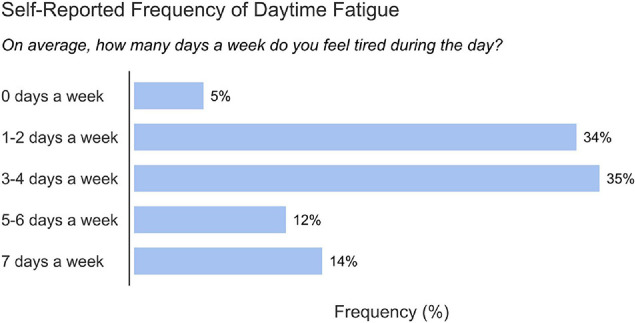
Bar plot showing the distribution of responses to the question about the average frequency of experiencing daytime fatigue.

The descriptive statistics for the scores obtained in the scales of the personality questionnaires are presented in Table 1. The percentage distribution of answers given to the question about the average frequency of experiencing daytime fatigue is depicted in Figure 1. Five percent of respondents in an average week do not feel tired during the day. About one-third of respondents (34%) indicated that they experience fatigue during the day on an average of 1–2 days per week. Another third of respondents (35%) reported feeling fatigued during the day an average of 3–4 days per week. 12 percent of respondents complained of feeling tired during the day most days of the week, and 14 percent experienced fatigue every day.

**TABLE 1:** Basic descriptive statistics for personality variables.

Variable	Mean	Limits of 95% confidence interval for mean		Median	Standard deviation	Interquartile range
Neuroticism	62.49	61.42	63.56	61.00	10.887	14
Volatility (N)	31.90	31.28	32.53	32.00	6.366	8
Withdrawal (N)	30.59	30.00	31.18	30.00	5.977	7
Agreeableness	71.11	70.05	72.16	71.00	10.694	16
Compassion (A)	36.10	35.45	36.75	36.00	6.566	9
Politeness (A)	35.01	34.46	35.55	35.00	5.558	8
Conscientiousness	64.28	63.36	65.20	63.00	9.344	13
Industriousness (C)	30.23	29.71	30.75	30.00	5.281	7
Orderliness (C)	34.05	33.49	34.61	34.00	5.696	8
Extraversion	64.65	63.66	65.63	64.00	10.026	12
Enthusiasm (E)	33.90	33.35	34.46	34.00	5.619	8
Assertiveness (E)	30.74	30.09	31.40	31.00	6.612	8
Openness/Intellect	66.67	65.75	67.59	65.00	9.370	12
Intellect (O)	32.75	32.25	33.25	32.00	5.124	7
Openness (O)	33.92	33.33	34.52	33.00	6.030	8
Self-esteem	32.88	32.36	33.41	33.00	5.313	6
Self-efficacy	29.65	29.25	30.04	30.00	4.015	4
Locus of control	5.54	5.25	5.84	6.00	3.033	5

The mean score in the Bedtime Procrastination Scale was equal to 3.372 points, with a standard deviation of 0.876 and a 95% confidence interval from 3.286 to 3.458. Results of the independent samples two-sided Welsh *t*-test indicated that the mean score in the Bedtime Procrastination Scale in the studied sample is significantly higher (*t* = 2.21; *df* = 671.98; *p* = 0.027) than in the Polish sample, which was drawn from the general population (Herzog-Krzywoszanska and Krzywoszanski, 2019), and the size of this difference is small (Cohen’s *d* = 0.17).

**FIGURE 2:**
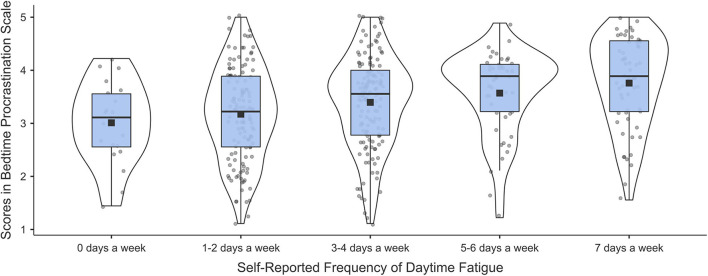
Violin plot showing the distribution of scores in the Bedtime Procrastination Scale at the different values of self-reported frequency of daytime fatigue. square points – means; bold horizontal lines – medians; box edges – lower and upper quartiles; the lower ends of the whiskers – minimal value or 1.5 interquartile range below lower quartile; the upper ends of the whiskers – maximal values; the violin lines – the probability densities of the scores in Bedtime Procrastination Scale, smoothed by a kernel density estimator; dots – jittered data points.

The frequency of experiencing daytime fatigue showed a weak, but significant positive correlation with the scores in the Bedtime Procrastination Scale (Spearman’s ρ = 0.26, *p* < 0.001). As can be seen at the violin plot presenting the distribution of scores in the Bedtime Procrastination Scale at the different values of self-reported frequency of daytime fatigue (see Figure 2.) the more often fatigue was experienced the higher scores in the Bedtime Procrastination Scale were.

**FIGURE 3:**
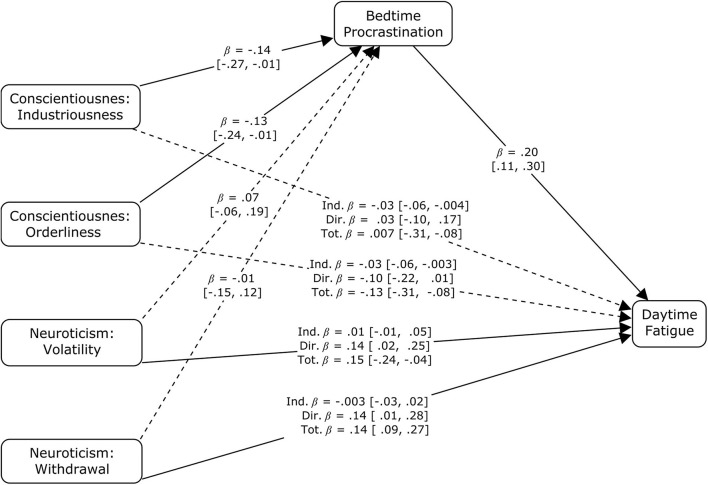
Standardized parameter estimates for indirect, direct, and total effects of the aspects of Conscientiousness and Neuroticism on self-reported average frequency of daytime fatigue with bedtime procrastination as a mediating variable. β – standardized estimate with the limits of bootstrapped 95% confidence intervals [given in brackets]; solid arrows – significant components of indirect effects or significant direct effects; dotted arrows – insignificant components of indirect effects or insignificant direct effects.

In the first mediation analysis, the model included the 5 personality dimensions from the Big Five model as predictors. The direct effect of scores on the Neuroticism scale (standardized estimate = 0.201 with bootstrapped 95% confidence interval from 0.099 to 0.305) and the indirect effect of scores on the Conscientiousness scale (standardized estimate = −0.053 with bootstrapped 95% confidence interval from −0.093 to −0.026) significantly differed from zero. Neither direct nor indirect effects were found for Extraversion, Agreeableness, and Openness/Intellect. Therefore, only scores for scales measuring both aspects of Neuroticism and Conscientiousness were entered into the second mediation model as predictors. As can be inferred from the limits of confidence intervals presented in Figure 3, both Volatility (standardized estimate = 0.136 with bootstrapped 95% confidence interval from 0.020 to 0.252) and Withdrawal (standardized estimate = 0.144 with bootstrapped 95% confidence interval from 0.009 to 0.280) directly impacted the self-reported averaged frequency of experiencing daytime fatigue. Industriousness (standardized estimate = −0.028 with bootstrapped 95% confidence interval from −0.062 to −0.004) and Orderliness (standardized estimate = −0.026 with bootstrapped 95% confidence interval from −0.058 to −0.003) scores were indirectly associated with daytime fatigue via the indirect path that leads through Bedtime Procrastination.

**FIGURE 4:**
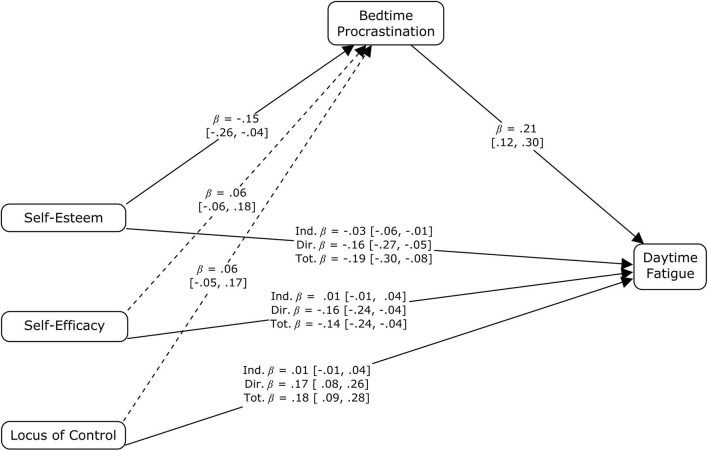
Standardized parameter estimates for indirect, direct and total effects of self-esteem, general self-efficacy and locus of control on self-reported average frequency of daytime fatigue with bedtime procrastination as a mediating variable. β – standardized estimate with the limits of bootstrapped 95% confidence intervals [given in brackets]; solid arrows – significant components of indirect effects or significant direct effects; dotted arrows – insignificant components of indirect effects.

The scores on the Rosenberg Self-Esteem Scale, the General Self-Efficacy Scale, and the Locus of Control scale of the Delta Questionnaire were entered in the third mediation model as predictors. The standardized parameter estimates for both the direct (standardized estimate = −0.163 with bootstrapped 95% confidence interval from −0.271 to −0.045) and indirect effects (standardized estimate = −0.031 with bootstrapped 95% confidence interval from −0.062 to −0.009) of self-esteem differed significantly from zero, whereas general self-efficacy (standardized estimate = −0.155 with bootstrapped 95% confidence interval from −0.253 to −0.056) and locus of control (standardized estimate = 0.169 with bootstrapped 95% confidence interval from 0.079 to 0.260) showed only a direct relationship with daytime fatigue (for details see Figure 4.).

## Discussion

The current study is the first to explore the relationship between bedtime procrastination and personality factors such as self-esteem, general self-efficacy, locus of control, and the basic personality traits as conceptualized in the Big Five Model. In our research, we tested whether bedtime procrastination helps to understand the relationship between personality characteristics and the outcome of sleep, namely fatigue.

### Conscientiousness and Fatigue

The results of our research have shown for the first time that bedtime procrastination mediates between both aspects of conscientiousness (industriousness and orderliness) and daytime fatigue. Industriousness was indirectly related to fatigue as a result of its negative effect on bedtime procrastination. This means that high industriousness reduces the level of bedtime procrastination, which in turn translates into decreased fatigue during the day. According to DeYoung et al. (2007), industriousness, which is proactive aspect of conscientiousness, prompts individuals to be persistent, self-disciplined, and to pursue achievement. People with high self-discipline can carry out tasks efficiently and on time, which means that they do not have to delay sleep to complete unfinished matters in the evening. On the other hand, people with a low level of industriousness due to poor self-discipline may have many duties left to perform in the evening, which means they postpone going to sleep to complete their planned activities. Additionally, their lack of self-discipline can mean that even if they manage to complete their obligatory tasks, they might procrastinate going to sleep to gain time because they cannot deny themselves pleasure, even if it involves fatigue the next day. As shown in previous studies, conscientious and persistent people more often pursue long-term goals, so they do not put momentary pleasure over their plans and ambitions, which could be hindered by reduced functioning during the day as a result of fatigue (Tedesqui and Young, 2018). Industrious people should also find it easier to overcome their aversion to a given task; this is considered one of the most important predictors of procrastination (Blunt and Pychyl, 2000) and is also associated with low conscientiousness (Watson, 2001). Given that sleep itself is generally not perceived as something unpleasant, aversion is supposed to involve interrupting pleasant activities (Kroese et al., 2014) or performing routine tasks that people usually do before bedtime, e.g., walking the dog or flossing teeth (Nauts et al., 2018). We may suspect that conscientious and self-disciplined individuals should be better able to overcome bedtime procrastination and thus go to bed early and sleep long enough to be refreshed.

Orderliness, which is the second aspect of conscientiousness, was also indirectly related to fatigue through bedtime procrastination. A high level of orderliness reduces the level of bedtime procrastination, which in turn is associated with a lower level of fatigue. Orderliness, as a hindering aspect of conscientiousness, refers to increased self-control, prudence, and accuracy (DeYoung et al., 2007). People with high levels of these traits find it easier to avoid bedtime procrastination because they can control their behavior and are therefore not inclined to impulsively engage in activities that delay going to bed (e.g., various kinds of entertainment). It is also possible that caution in such people makes them more predictive than people who score lower on the orderliness scale, making them more aware of the consequences of their actions and the effects of bedtime procrastination on their fatigue the next day. They may also be motivated by the need to perform tasks accurately and reliably, which is less likely when an individual is sleepy and tired.

The obtained results can also be explained by the health impact of bedtime procrastination. It has been proven that conscientious people care more about their health (Booth-Kewley and Vickers, 1994; Ingledew and Brunning, 1999), and it is commonly known that adequate sleep duration is an important factor in proper psychophysical functioning. One’s concern for one’s own health may result from a high level of orderliness and related prudence. Therefore, we suppose that taking care of their health is an additional motivation for people who have the habit of going to bed at the right time, which results in general well-being the next day and a feeling of being well-rested.

### Neuroticism and Fatigue

Convergently with the results of previous studies (Mastin et al., 2005), the results of our research show that neuroticism as well as volatility and withdrawal, both of which are aspects of neuroticism, were found to be directly and positively related to daytime fatigue without the intermediary impact of bedtime procrastination. To the best of our knowledge, the current study shows for the first time that both aspects of neuroticism (volatility and withdrawal) are related to daytime fatigue. Both high volatility (including irritability, emotional lability, and poor control of negative emotional reactions) and high withdrawal (defined as a tendency to feel anxious and worried) (DeYoung et al., 2007) are associated with more frequent experience of daytime fatigue during the week. We found that bedtime procrastination did not mediate the relationship between neuroticism and daytime fatigue. Thus, some factors other than bedtime procrastination may substantially mediate the negative impact of neuroticism on sleep duration and quality, as well as the resulting daytime fatigue. Mainly due to their negative affectivity, neurotic people possibly feel tired because of difficulty falling asleep and generally worse quality of sleep. Neurotic people’s frequent negative emotions may directly worsen their sleep outcomes (DeYoung et al., 2007; Slavish et al., 2018).

One of the reasons for neurotic people’s sleep difficulties may be their tendency to ruminate (Thorsteinsson et al., 2019), which is associated with higher impulsivity. As shown by various studies, impulsivity positively correlates with both rumination and sleep dysfunction; this may be due to the inability to suppress negative thoughts, which can make it difficult to fall asleep (Lozano et al., 2014). It has also been shown that high impulsivity is associated with an increased frequency of nightmares and difficulties in maintaining sleep (Schmidt et al., 2008), which can also induce daytime fatigue. Highly impulsive people may also experience daytime fatigue more often because it takes much more mental effort for them to achieve similar study or work results than less impulsive people. It has been proven that impulsive students achieve worse academic results (Lozano et al., 2014); impulsivity is also negatively correlated with reasoning, probably because high impulsivity impairs the ability to reason in some way (Schweizer, 2002). It is also noteworthy that depression and anxiety are associated with neuroticism and fatigue (Kato et al., 2006; Poeschla et al., 2013; Vassend et al., 2018). The daytime fatigue experienced by neurotic people is possibly caused by frequent distress, worry, and focusing on negative feelings, but it does not result from a deficiency of sleep at night due to bedtime procrastination.

### Self-Esteem and Fatigue

Bedtime procrastination mediates the relationship between self-esteem and fatigue. Moreover, self-esteem is also directly related to fatigue. This means that low self-esteem is associated with high bedtime procrastination, which consequently leads to increased fatigue. In addition, low self-esteem also directly contributes to fatigue. One possible explanation for these results could be self-handicapping through bedtime procrastination by people with uncertain self-esteem. Delaying a task until the last minute allows them to blame shortcomings in their work on a lack of time instead of a lack of capacity, which protects self-esteem (Steel, 2007). Bedtime procrastination can also be a form of self-handicapping (Strunk and Steele, 2011). The consequence of delaying bedtime is often fatigue and lack of sleep the next day. Possible failure to complete demanding tasks may be explained by fatigue and sleep deprivation, which again protects self-esteem. If self-handicapping results in poor performance, then failure may be attributed to a handicap or obstacle and not a personal lack of ability.

Another possible explanation for the obtained results takes into account the fact that self-esteem may often fluctuate as a result of successes and failures (Cast and Burke, 2002). Successful completion of all tasks planned for a day can be interpreted as a success, but incomplete tasks are regarded as a failure. So, it is likely that people try to finish all tasks started on a given day in order to protect their self-esteem. If the planned activities drag on until late in the evening, there may be no time left to rest before bedtime. Then, instead of going to sleep, the individual engages in pleasant activities, e.g., watching movies. Anderson (2016) believes that in certain situations people allow themselves to procrastinate because they feel that they have worked hard and they deserve a rest, even if it means delaying bedtime and feeling tired the next day. This may be an attempt to avoid guilt and protect self-esteem.

Our research also found an association between low self-esteem and fatigue without mediation by sleep procrastination. Low or unstable self-esteem is often associated with a tendency to feel negative emotions and low mood, which in turn are often accompanied by fatigue (Franck and De Raedt, 2007). As shown by Lemola et al. (2013), there is a relationship between low self-esteem and insomnia. It is possible that people with low self-esteem do not get enough sleep and therefore become tired, but this is not because of bedtime procrastination.

### Self-Efficacy and Fatigue

Our study found that low self-efficacy was associated with increased daytime fatigue without the intermediary role of bedtime procrastination. This can be explained by the lower ability of people with low self-efficacy to adapt to environmental requirements in order to cope with stress (Burger and Samuel, 2017). General self-efficacy is associated with people’s belief in their ability to cope effectively even with ambiguous and stressful tasks (Cast and Burke, 2002). This belief seems to be particularly important for university students, who face numerous changes, challenges, encumbrances, new roles, and social and financial problems. Difficult situations can be a source of stress that negatively affects psychophysiological health, including increased fatigue (Walters and Denton, 1997; Kocalevent et al., 2011). The impact of potential stressors depends on intrapersonal resources (Folkman and Lazarus, 1988), including self-efficacy and self-esteem. Several studies have shown that self-efficacy is a positive predictor of adaptive stress coping in students (Burger and Samuel, 2017), which suggests that fatigue may be higher in students with lower self-efficacy due to the negative emotional consequences of poor coping, which may worsen the quality of night sleep. Another explanation for the results obtained in our research may be the relationship between self-efficacy and the effectiveness of implementation of tasks. People with high self-efficacy more often plan their activities (Gollwitzer, 1999), which improves the implementation of tasks and the achievement of goals (Schunk, 1995). Low efficiency of activities may result in slower performance of tasks, accumulation of duties and, consequently, greater fatigue.

### Locus of Control and Fatigue

Our results showed that bedtime procrastination does not mediate the relationship between locus of control and fatigue. External locus of control was directly related to daytime fatigue without being mediated by procrastination. The direct link between locus of control and fatigue may be explained by externals’ greater sensitivity to sleep deprivation. The studies by Kumar and Vaidya (1986) revealed that externals usually sleep significantly longer than internals, which may reflect their increased sleep needs. This assumption seems to be confirmed by the results of the studies by Hill et al. (1996), which showed that externals experienced a greater increase in fatigue due to sleep deprivation than people with an internal locus of control. An alternative explanation for our results may be the increased susceptibility of externals to experiencing stress and anxiety. Previous studies showed that externals have a higher level of stress and arousal than internals (Anderson, 1976). Since a high level of perceived stress and arousal negatively affects sleep quality (Knudsen et al., 2007), fatigue in externals may be greater than in internals.

### Limitations and Future Directions

The average score in the Bedtime Procrastination Scale was higher in the studied sample of university students than in the Polish sample, which was drawn from the general population (Herzog-Krzywoszanska and Krzywoszanski, 2019). This difference is in line with the results of the previous studies (Kang and Chen, 2009; Cheng et al., 2012) that report irregular sleeping habits, poor sleep quality and a high incidence of sleep problems in students. The rationale for conducting the study on a sample of university students was to include those at the highest risk for poor sleep habits, shortened sleep length, and reduced sleep quality. However, the view of the analyzed relationships obtained from the results of this study may be incomplete or biased. Therefore, further research on the role of personality in sleep problems should also include subjects from other social groups and incorporate demographic characteristics in modeling the results.

Another limitation of our study was the use of only self-report questionnaire research tools that only measured subjective aspects of sleep problems. The important direction for further research on the psychosocial determinants of sleep deficit, sleep latency, and other sleep problems should be to expand the range of variables analyzed to include more quantitative measures of sleep pattern and quality, recorded with mobile wearable devices or polysomnographic techniques.

Furthermore, this study focused only on the most evident unidirectional relationship between bedtime behavior and daytime fatigue and did not consider the possibility of bidirectional relationships between these variables. Given the difficulty of capturing such bidirectional relationships in cross-sectional studies, the use of longitudinal design should be considered in further research.

## Conclusion

Our research revealed new insights into the mediating role of bedtime procrastination in the relationship between personality characteristics and daytime fatigue. Identifying the personality traits that influence sleep outcomes and the role of bedtime procrastination as a mediating factor provides better insight into the possible causes of fatigue. It also indicates new directions for possible therapeutic interventions. The results obtained can help in tailoring prevention and intervention strategies to the individual characteristics of people who do not go to bed on time and consequently experience daytime fatigue. The effectiveness of interventions that aim to improve sleep-related behaviors and reduce fatigue in people can be increased by taking into account personality sources and the individual specificity of their problems.

The fact that low conscientiousness and low self-esteem is associated with greater fatigue indirectly through bedtime procrastination suggests that people with daytime fatigue may benefit from using some methods of self-regulation improvement. Self-regulatory skills training includes stimulus control techniques, goal definition techniques, and time management techniques (Eerde and Klingsieck, 2018). People with low conscientiousness or low self-esteem should be taught specific strategies, such as planning, prioritizing, and monitoring progress. These tools would allow them to reduce bedtime procrastination; consequently, their daytime fatigue would be reduced and their satisfaction with life would increase. In turn, high neuroticism is associated with greater fatigue without the mediation of bedtime procrastination. Neurotics are prone to sudden and disproportionate arousal due to emotional stimuli and to a slow decline of arousal (Eysenck and Eysenck, 1985), which is associated with increased susceptibility to stress (Suls, 2001). Bader et al. (2011) found that exposure to stressful videos leads to worse sleep, especially if it activates memories of stressful life events. Mindfulness is a method that is helpful in reducing neuroticism and stress, and it also improves sleep quality (Cincotta et al., 2011; Oken et al., 2014; Armstrong and Rimes, 2016). Since even a brief mindfulness intervention improves sleep quality and increases the feeling of being refreshed (Hülsheger and Schewe, 2011; Li et al., 2018), it seems to be worth applying to people experiencing daytime fatigue as a result of bedtime procrastination.

Since negative affectivity is an important factor of neuroticism that affects sleep, strategies for reducing negative mood and increasing positive one may be an effective way to help reduce daytime fatigue (Latif et al., 2019). These include techniques for regulating emotions (e.g., positive reappraisal) or improving well-being through positive interventions (e.g., gratitude) and by developing mental resources. The research by Ng (2016) showed that the level of happiness in people with high neuroticism who applied a positive intervention that consisted of occasionally imagining the best version of themselves was higher than in people in the control group. In their studies, Latif et al. (2019) showed that positive affect was positively correlated with sleep quality, while negative affect and suppression of feelings were negatively correlated with sleep quality. It has been suggested that reducing negative affectivity and treating symptoms of depression and anxiety may enhance the effectiveness of psychological interventions for sleep problems (Stewart et al., 2011).

The results of our research indicate that to reduce fatigue in people, emphasis should also be placed on improving their self-esteem, increasing their self-efficacy, and enhancing their expectations of control over their lives. For this purpose, one can use instruction-delivery techniques or effort-strengthening techniques; shaping positive beliefs about one’s abilities and facilitating a feeling of pride and satisfaction with personal efforts are also effective (Abraham and Michie, 2008).

In addition, certain personality characteristics, such as high neuroticism, low conscientiousness, low self-esteem, low self-efficiency, and external locus of control, can help identify people with the highest risk of experiencing daytime fatigue; therefore, steps can be taken to prevent them from developing sleep problems.

## Data Availability Statement

The raw data supporting the conclusions of this article will be made available by the authors, without undue reservation.

## Ethics Statement

The studies involving human participants were reviewed and approved by Faculty Committee for Research Ethics, Faculty of Pedagogy, Pedagogical University of Kraków. The patients/participants provided their written informed consent to participate in this study.

## Author Contributions

RH-K and BJ contributed to the concept and the study design. RH-K and LK organized and conducted the research. LK performed the statistical analysis and prepared the Figures. All authors wrote the manuscript, contributed to the revision of the manuscript, and read and approved the submitted version.

## Conflict of Interest

The authors declare that the research was conducted in the absence of any commercial or financial relationships that could be construed as a potential conflict of interest.

## Publisher’s Note

All claims expressed in this article are solely those of the authors and do not necessarily represent those of their affiliated organizations, or those of the publisher, the editors and the reviewers. Any product that may be evaluated in this article, or claim that may be made by its manufacturer, is not guaranteed or endorsed by the publisher.
